# Characterization of a human powered nebulizer compressor for resource poor settings

**DOI:** 10.1186/1475-925X-13-77

**Published:** 2014-06-16

**Authors:** Christopher J Hallberg, Mary Therese Lysaught, Christopher E Zmudka, William K Kopesky, Lars E Olson

**Affiliations:** 1 Department of Biomedical Engineering, Marquette University, Milwaukee, WI, USA; 2 Institute of Pastoral Studies, Loyola University, Chicago, IL, USA; 3 Neiswanger Institute for Bioethics & Health Policy, Stritch School of Medicine, Loyola University, Chicago, IL, USA; 4 School of Medicine, University of Washington, Seattle, WA, USA; 5 Particle Technology Labs, Downers Grove, IL, USA

## Abstract

**Background:**

Respiratory disease accounts for three of the ten leading causes of death worldwide. Many of these diseases can be treated and diagnosed using a nebulizer. Nebulizers can also be used to safely and efficiently deliver vaccines. Unfortunately, commercially available nebulizers are not designed for use in regions of the world where lung disease is most prevalent: they are electricity-dependent, cost-prohibitive, and not built to be reliable in harsh operating conditions or under frequent use.

To overcome these limitations, the Human Powered Nebulizer compressor (HPN) was developed. The HPN does not require electricity; instead airflow is generated manually through a hand-crank or bicycle-style pedal system. A health care worker or other trained individual operates the device while the patient receives treatment.

This study demonstrates functional specifications of the HPN in comparison with a standard commercially available electric jet nebulizer compressor, the DeVilbiss Pulmo-Aide 5650D (Pulmo-Aide).

**Methods:**

Pressure and flow characteristics were measured with a rotameter and pressure transducer, respectively. Volume nebulized by each compressor was determined by mass, and particle size distribution was determined via laser diffraction. The Hudson RCI Micro Mist nebulizer mouthpiece was used with both compressors.

**Results:**

The pressure and flow generated by the HPN and Pulmo-Aide were: 15.17 psi and 10.5 L/min; and 14.65 psi and 11.2 L/min, respectively. The volume of liquid delivered by each was equivalent, 1.097 ± 0.107 mL (mean ± s.e.m., n = 13) for the HPN and 1.092 ± 0.116 mL for the Pulmo-Aide. The average particle size was also equivalent, 5.38 ± 0.040 micrometers (mean ± s.e.m., n = 7) and 5.40 ± 0.025 micrometers, respectively.

**Conclusions:**

Based on these characteristics, the HPN’s performance is equivalent to a popular commercially available electric nebulizer compressor. The findings presented in this paper, combined with the results of two published clinical studies, suggest that the HPN could serve as an important diagnostic and therapeutic tool in the fight against global respiratory health challenges including: tuberculosis, chronic obstructive pulmonary disease, asthma, and lower respiratory infections.

## Background

Respiratory disease is a leading cause of death worldwide. According to the World Health Organization, lower respiratory infections (LRIs), chronic obstructive pulmonary disease (COPD), and tuberculosis (TB) are major causes of mortality, ranking 3rd, 4th, and 11th respectively, killing 3.2, 3 and 1 million persons in 2011 [1]. In low-income countries, LRIs are the leading killer and usually strike children under 5 years of age. Some estimate that in 2011, 64 million people suffered from COPD worldwide [2], with a profound effect on death and disability [3]. TB is a leading cause of death among HIV-positive individuals and is co-epidemic with HIV in sub-Saharan Africa [4]. By any measure, these three diseases are grave global health challenges.

At first glance, these three diseases share only the organ they impact. Etiology, diagnosis, treatment, and prevention strategies for these conditions are diverse in nature and technology. In addition to being diseases of the lung, they do share the populations they impact, namely the poor throughout low and middle-income countries, particularly in regions without access to primary health care. Many facets of poverty augment the incidence and mortality of these conditions. Health care for the poor is confounded by a lack of infrastructure and a dearth of healthcare providers. These factors suggest a need for a simple, appropriate solution, ideally a single technology platform that can address all these diseases.

Direct delivery of therapeutics to the lung tissue has long been a successful strategy [5]. Inhaled aerosolized medicine targets the lung tissue while minimizing systemic side effects and has the potential to require less medicine. Technologies for direct delivery include various types of inhalers and nebulizers. The metered dose inhaler (MDI) is a standard technology for bronchodilator treatment of reactive airway diseases, like COPD and asthma. Dry powder inhalers (DPIs) have also been used to deliver a variety of drugs and are advantageous in some situations.

Nebulizers have been used widely as well [6]. They have been used in the administration of bronchodilators and antibiotic delivery [5,7-9]. In TB diagnosis, nebulized hypertonic saline is a successful way to obtain sputum samples for smear tests and other tests in individuals, especially as part of intensified case-finding in those who do not yet have a productive cough [10]. Intriguingly, there is evidence that a new potential treatment for certain LRIs using nebulized hypertonic saline may prove effective [11,12]. Nebulized vaccines, including the measles and measles-mumps-rubella vaccines, have been shown to produce an equivalent, and in some studies stronger, immune response than their injected counterparts, without the associated risk of blood-borne disease transmission [13,14].

Each of these delivery technologies has advantages and disadvantages. MDIs are small and portable, but their effectiveness has been questioned because of problems in patient inhaler technique and the need for frequent re-training [15,16]. This drawback could be amplified in regions where health professionals are already overburdened and unable to provide requisite training to patients. Use of valved spacers with MDIs lessens the importance of proper technique and improves lung deposition [16]. Spacers, however, represent an additional cost and require periodic cleaning to remain functional, which lessen their attractiveness for use in resource poor settings. DPIs use the patient's inhalatory force to deliver medicines to the lower respiratory tract obviating the need for propellants. DPIs have noted limitations with regard to delivery system reliability and require special drug formulations to create the ideal particle diameter [17]. Also inhalers, both DPIs and MDIs, are limited in the volume they can deliver and could never be used for certain applications, such as delivery of hypertonic saline solution, for example. Moreover, inhaler devices, particularly DPIs, each have different operating instructions, requiring significant investment in training by the implementing body for each of these technologies.

The nebulizer, however, could provide a single platform for diagnosing TB, treating COPD and LRIs, and delivering vaccines. Jet nebulizers have several distinct advantages over other aerosolized technologies: improved patient compliance, less patient training, and potentially lower per-treatment costs. Jet nebulizers have the benefit of being less likely to denature the therapeutics they deliver, in contrast to ultrasonic nebulizers [18,19].

With respect to DPIs and MDIs, nebulizers have the ability to deliver substantial volumes of liquid treatments. The chief drawback of nebulizers is that they are less portable. Ultrasonic and vibrating mesh nebulizers are portable, but they are more expensive, have components that require more frequent replacement, and are not intended for continuous use, such as a clinical setting [19]. In other words, they may not be particularly suited for developing contexts.

A key limitation of jet nebulizers in developing contexts is that they require significant amounts of electric power, on the order of 100 W. Regions where electricity is non-existent or unreliable experience increased incidence of respiratory diseases, for example, those caused by indoor air pollution [20,21]. Ultrasonic and vibrating mesh nebulizers require less power than jet nebulizers, but their higher cost and need for battery recharging still exists. One reason why the jet nebulizer as a single-platform-multiple-use concept has not been explored in global respiratory health may be this requirement for electricity.

The Human Powered Nebulizer (HPN) was invented to address this problem. The HPN is a manual compressor that works with existing, inexpensive nebulizer mouthpieces. The purpose of this study was to compare the performance of the HPN to an electric nebulizer compressor on key performance measures: volume delivery, pressure and flow characteristics, and delivered particle size distribution. The present study complements two published clinical trials that have demonstrated equivalence between the HPN and an electric nebulizer for the production of sputum samples for TB diagnosis [22] and for the treatment of patients with mild to moderate asthma exacerbations [23].

## Methods

Key to the administration of liquid medication deep into the lungs is the particle size distribution of aerosol particles coming from the nebulizer. A range of 1–5 microns in particle diameter has been suggested as maximizing deposition deep into the lungs [6]. The nebulizer mouthpiece used in this study, the Hudson RCI Micro Mist, makes an aerosol from a jet of air that flows through a submersed orifice. Compared to other nebulizer mouthpieces, the output of the Micro Mist is relatively insensitive to changes in the angle of the mouthpiece, thus requiring less training. To ensure a consistent particle size distribution for this and many other nebulizer mouthpieces of this type, the velocity of air past the submersed orifice must be within a certain range. Therefore, the key design parameter of the jet nebulizer compressor is a constant specific airflow at a pressure sufficient to overcome the hydraulic resistance of the nebulizer mouthpiece itself.

### Design

**Figure 1:**
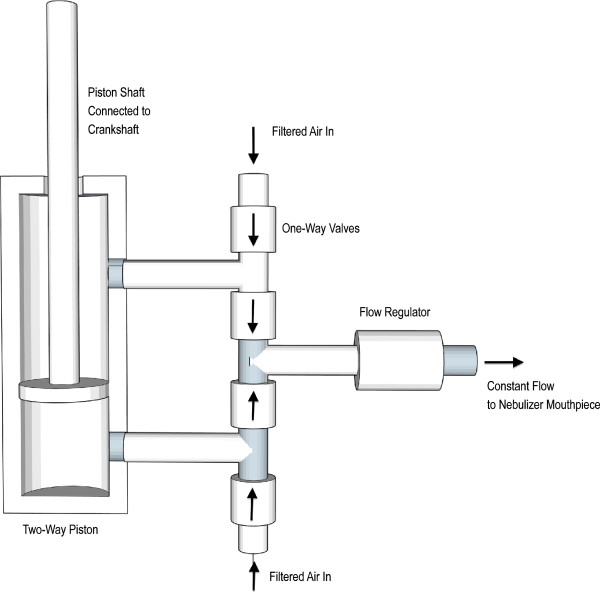
**Airflow schematic.** Internal airflow mechanism of the HPN is shown. One-way check valves, direction of flow indicated by arrows, dictate the unidirectional flow for the pistons, represented by diagonal line shading. Pistons are bi-directional and, for the two-piston configuration, positioned 90° out of phase to generate 4 piston cycles per revolution of the crankshaft connected to the pistons. The piston output through a flow regulator, shown on the right.

The basic design of the HPN creates airflow and pressure using pistons, model 042-DXPE (Bimba Manufacturing, Monee, IL, USA) that are driven by human power. In our designs, dual-action pistons were used so that airflow would be generated during the entire piston cycle. The configuration for the airflow system is shown in Figure 1.

Each piston has two inlet/outlets. A tee is connected to each inlet/outlet. On one end of the tee is a one-way valve, model 64048 (US Plastics, Lima, OH, USA) directed inward so that air can flow into the piston. The other end of the tee is connected to a one-way valve directed outward so that air can flow from the piston to the nebulizer mouthpiece. Connections were made with Tygon® tubing, model 5114 K54 (US Plastics, Lima, OH, USA). This outlet flow was directed through a flow regulator, model Y1-4 NPT (Gate LLC, Sebastian, FL, USA). The outflow from the flow regulator was then delivered to the nebulizer mouthpiece via the tubing supplied by the mouthpiece manufacturer. In order to generate the requisite flow rate using human power with the aforementioned pistons, gearing systems were selected to achieve an effective frequency of piston operation with minimal human exertion.

**Figure 2:**
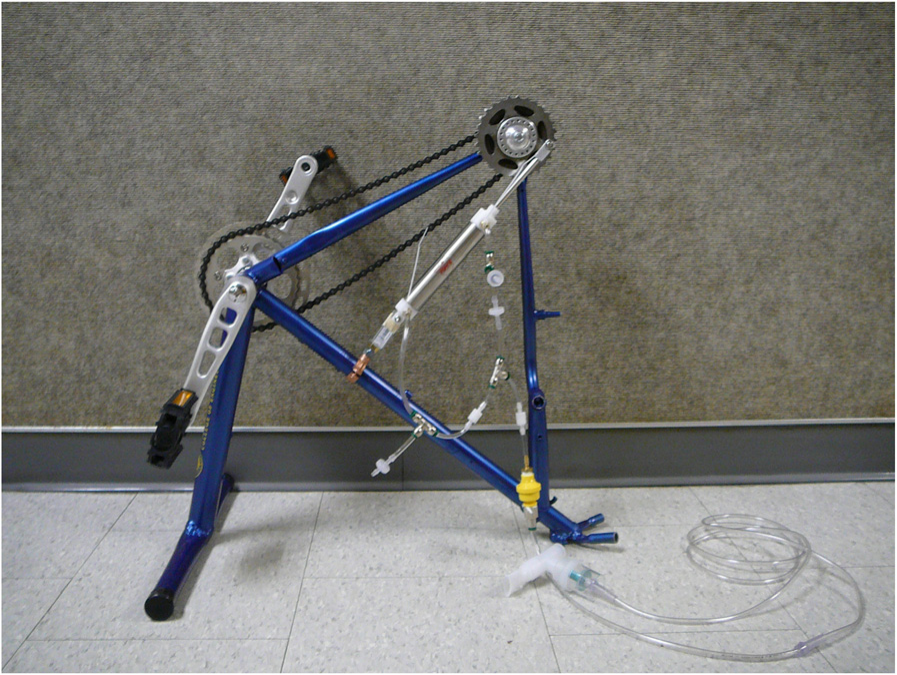
**Bicycle compressor.** The bicycle design of the HPN is a stationary single bi-directional piston design. This prototype device was constructed from a commercially available bicycle frame. The frame was cut and re-welded to produce the device. The silver cylinder is a bi-directional piston attached to the frame and the gear. The white plastic pieces are the one-way valves configured in the manner indicated in Figure 1, and the yellow plastic piece is the flow regulator. The nebulizer mouthpiece and tubing is connected to the output of the flow regulator. The operator—who is someone other than the patient—sits in a chair and pedals the device by foot. The effort required is roughly equivalent to riding a bicycle at a rate of eight miles per hour.

**Figure 3:**
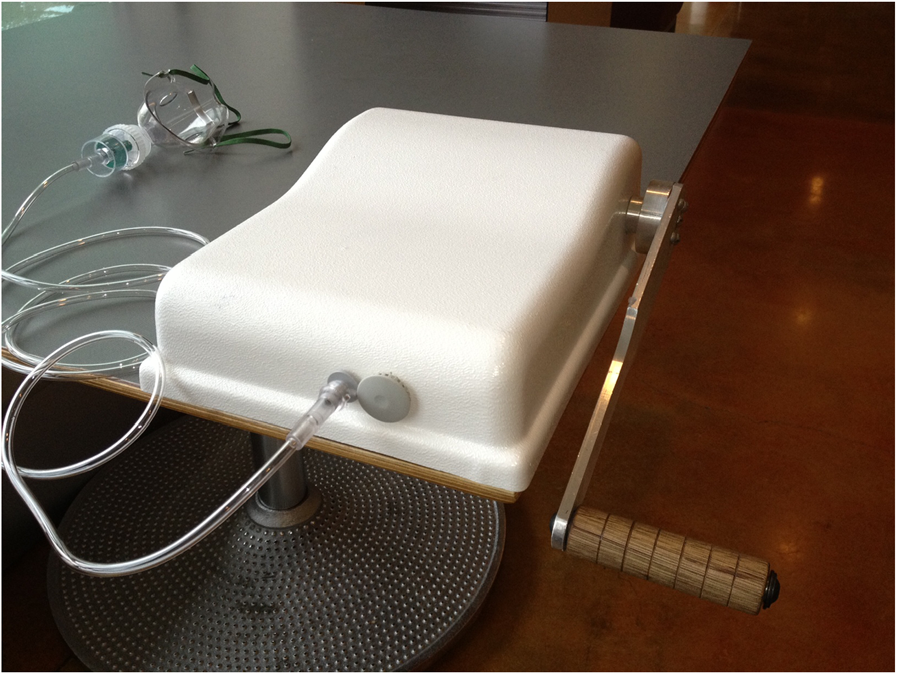
**Hand-powered compressor.** The hand-cranked version of the HPN is a sealed unit that can be operated by a health worker on a table-top. The pneumatic configuration which was given in Figure 1 is not shown, but is similar to that in Figure 2. The gray disk to the right houses the filter for the nebulizer mouthpiece and the adaptor on the left is connected to the neublizer mouthpiece. The hand crank on the right can be cranked in either direction by someone other than the patient. The hand cranked design emerged through conversations with health workers who desired a device that could be more readily transported. Additionally, a table-top device was preferred over a device operated on the floor. In many regions of the world people grind corn with hand operated mills of a similar design. This device can be carried in a backpack with room for supplies.

Two HPN designs were studied: a bicycle-based pedal version using one dual-action piston, the functional equivalent of two pistons, and a hand-cranked version using two dual-action pistons, the functional equivalent of four effective pistons. See Figures 2 and 3. For each version, a healthcare provider would pedal or crank at approximately one revolution per second.

The bicycle version uses a standard bottom bracket pedal combination, gears and a chain connected to a modified bicycle frame that has been welded so as to secure the pistons in a stationary position. The length of the pedal crank was 18 cm, attached to a 36-tooth gear which drove a 13-tooth gear attached to a modified wheel hub, for a gear ratio of approximately 1:3 – every full turn of the pedal generated 3 piston strokes. A 5 cm plate was attached to the hub, and connected to the piston with a pin. This limited the travel of the piston, i.e., the effective lever arm on the piston, to 10 cm.

The hand-cranked version uses a direct gear system, with a 22 cm long handle connected to a two-gear system, models S1VS24-100 F1524 and S1VS24-025 F1516 (SDP/SI, New Hyde Park, NY, USA). The gears were connected to a custom-built crankshaft which then connected to the pistons. The gear ratio in this version was 1:4. For both the bicycle and hand cranked version, the force needed to operate the HPN was approximately 18 N, the equivalent energy expenditure required to pedal a bicycle at approximately 8 miles/hour.

### Measurements

In all comparison studies in this work, the HPN was compared to a DeVilbiss Pulmo-Aide 5650D compressor (DeVilbiss, Somerset, PA, USA). The nebulizer mouthpiece used was the Hudson RCI Micro Mist (Teleflex, Research Triangle Park, NC, USA). All experiments were conducted between 20 – 24°C at relative humidity between 50 and 60%.

#### Pressure-flow measurements

Two configurations were used for measuring pressure and flow. In the first configuration, airflow was measured using a variable area rotameter, model MR3000-30 lpm (Key Instruments, Trevose, PA, USA) without the nebulizer mouthpiece in place. The outflow of the rotameter was open to atmospheric pressure. The output of the nebulizer compressors was connected to the rotameter with Tygon tubing. In the second configuration, upstream pressure was measured using a pressure transducer, model PX139-03004 V (Omega Engineering, Stamford, CT, USA) with output pressure range between 0 and 30 psi. The pressure transducer output was collected using an A/D converter, model NI USB-6008 (National Instruments, Austin, TX, USA) and stored on a computer. Pressure was measured both with and without the nebulizer mouthpiece downstream in the airflow circuit. The nebulizer mouthpiece was connected to the nebulizer compressor with a 120 cm length of Tygon tubing. The tubing was cut half the distance between the mouthpiece and the compressor and a plastic tee was inserted. The third branch of the tee was connected to the pressure transducer with a 15 cm piece of Tygon tubing.

#### Volume nebulized measurements

For these experiments, 5 mL of water was placed in the nebulizer mouthpiece cup and weighed. The nebulizer compressor under test was operated for 5 minutes. The mouthpiece was held stationary, in a vertical position, for the duration of the test. After the operating period, the nebulizer mouthpiece was weighed again and the difference in mass and, thus, volume was recorded.

#### Particle size distribution measurements

Particle size distribution was measured using the hand-crank HPN design and the DeVilbiss compressor with a Malvern Mastersizer S laser diffraction particle size analyzer (Malvern Instruments Ltd, Malvern, UK). The output of the Micro Mist nebulizer mouthpiece was positioned 2.5 cm from the laser beam of the Mastersizer. The outflow of the mouthpiece was oriented vertically so that the narrowest part of the mist interacted with the laser light. Without the force of a patient’s inspiratory flow, the output of the nebulizer mouthpiece formed a swirling cloud of particles near the output of the mouthpiece of the device. To accurately determine particle size, air from a tank was passed through one end of the mouthpiece at 15 L/min to propagate the mist across the laser path. Particle size was determined with both 2.5 and 5 mL volumes of normal saline in each of the mouthpieces to determine the effect of fill volume on particle distribution. Particle size distribution using either the HPN or the DeVilbiss compressor was recorded. The Mastersizer reports droplet size in volume median (Dv0.5) values: the diameter of the delivered particle at which 50% is smaller than the indicated size. Dv0.5 can be assumed to be equivalent to the mean mass aerodynamic diameter (MMAD) determined by cascade impactor particle sizing if the density of particles is constant across the particle distribution, as is the case in this study. For ease of comparison to other values in the literature, particle size will be reported as MMAD.

## Results

**Figure 4:**
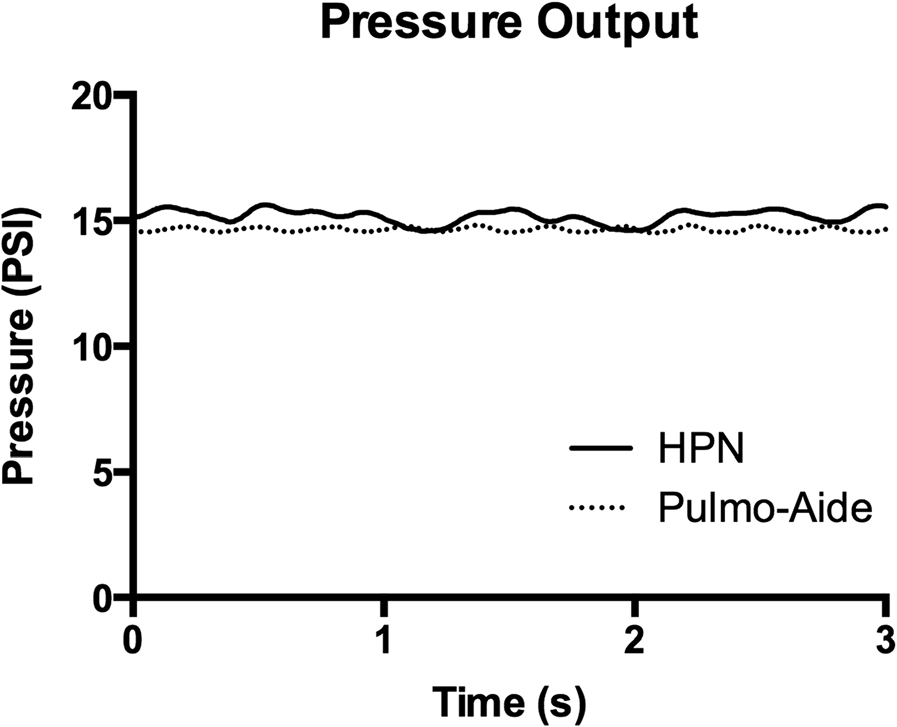
**Pressure.** Pressure versus time for the HPN shows equivalence. The difference between the two is below the variation seen among different nebulizer compressors.

The volume nebulized during a five minute period for the HPN and Pulmo-Aide was: 1.097 ± 0.107 mL (mean ± s.e.m, n = 17), and 1.092 ± 0.116 mL, respectively. Average pressure sampled over four seconds for the HPN and Pulmo-Aide was: 15.17 ± 0.010 psi (mean ± s.e.m) and 14.65 ± 0.001 psi, respectively. Figure 4 shows the pressure data for the two-piston, hand-cranked HPN and the Pulmo-Aide. Free flow rate for the HPN and Pulmo-Aide was: 10.5 L/min and 11.2 L/m, respectively.

**Figure 5:**
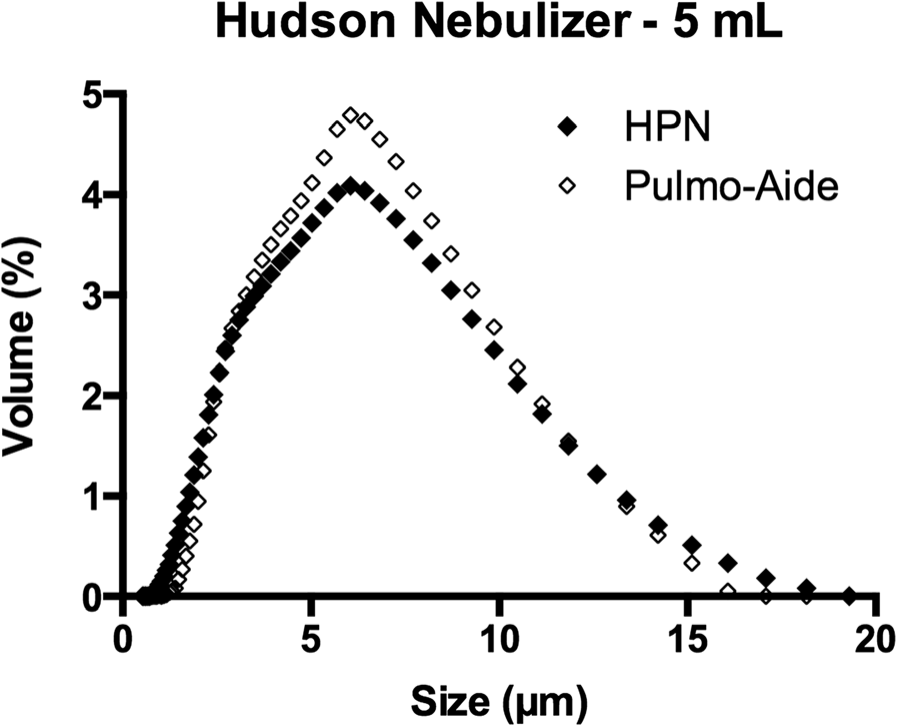
**Particle size distribution – 5.0 mL.** Particle size distribution for HPN and Pulmo-Aide nebulizers with 5.0 mL of saline in nebulizer cup is shown.

**Figure 6:**
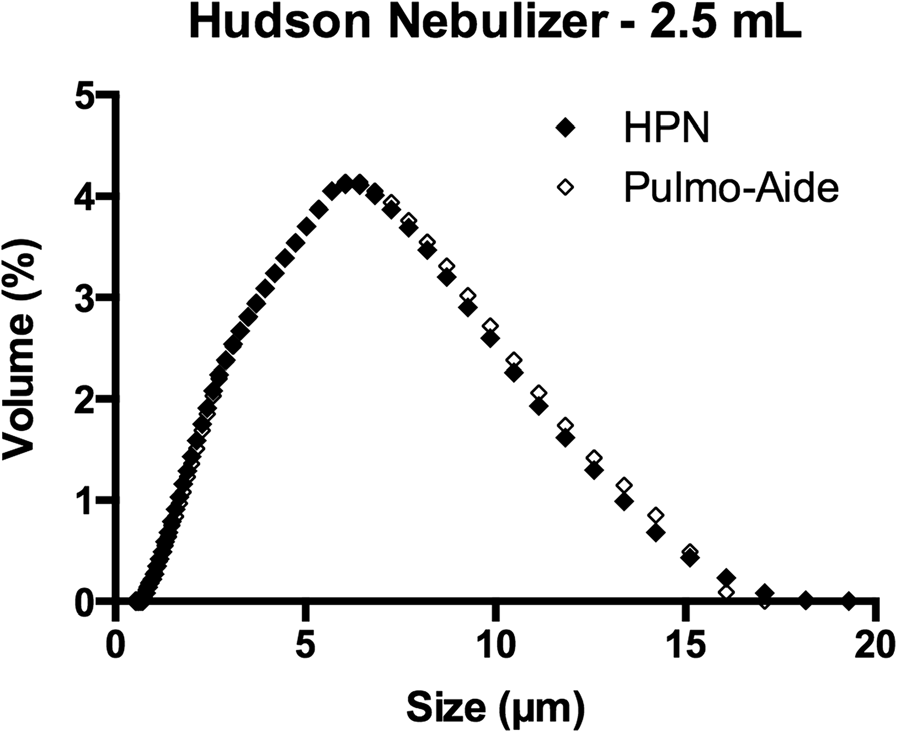
**Particle size distribution – 2.5 mL.** Particle size distribution for HPN and Pulmo-Aide nebulizers with 2.5 mL of saline in nebulizer cup is shown.

Shown in Table 1 are the results of the particle sizing experiments. In it are shown the MMAD in micrometers as mean ± s.e.m., n = 7. Fill volume had no effect on particle size distribution. Figures 5 and 6 contain particle distributions for each fill volume.

**Table 1:** Particle size

	**Nebulizer fill volume**	
	**5.0 mL**	**2.5 mL**
HPN	5.38 ± 0.040	5.36 ± 0.058
Pulmo-Aide	5.40 ± 0.025	5.42 ± 0.016

MMAD values in micrometers. Nebulizer mouthpiece was Hudson RCI Micro Mist.

## Discussion

The idea of a nebulizer designed for rural or remote regions is not new. The classic Mexican device for aerosolized vaccine delivery is perhaps the best known [13]. One physician who was practicing medicine in El Salvador during its civil war in the 1980’s developed a device based on a large syringe-like pump (personal communication with Fausto Cea Gil, M.D., San Salvador, 2010). The initial HPN prototypes were also simple, and lacked necessary design features that make the HPN equivalent to the Pulmo-Aide model in an effort to keep the design affordable. Other hand-powered devices have also been manufactured; DeVilbiss offers a nebulizer mouthpiece driven by a squeeze bulb. Although it generates particles of respirable size, 5 μm MMAD, the rate of volume delivery is far less than modern electric compressors. Cameron-Price manufactures a foot pump nebulizer compressor with output characteristics similar to the Pulmo-Aide when operated properly. However, this model does not have a flow regulator to ensure proper flow rate and as a result, high variability in performance is likely [24].

The goal of the HPN project and of this study was to design a nebulizer compressor that was low cost. The project also aimed to develop a device that conformed to a commonly-used commercially available electric model with typical output characteristics. The hypothesis of the study presented here was that the HPN would perform equivalent to a commercially available electric nebulizer compressor, and the evidence from this study consistently supports that hypothesis. Based on the data presented here, the HPN performs equivalently to the Pulmo-Aide, which was selected for comparison in our study because it is a commonly used, well characterized, compressor model.

The small differences in flow and pressure values are well within range of other commercially available nebulizer compressors. Smith et al. presented a performance comparison of 23 different models of jet nebulizer compressors [24]. In this study, the mean particle diameter varied from 2.6 to 10.2 micrometers. Flow ranged from 3 to 8 L/min, and the volume of drug delivery also varied quite widely. This study is instructive in the design of the current work in two ways. First, the HPN functions within the particle size range offered by electric compressors and nebulizer mouthpiece pairings evaluated by Smith et al. Second, the variance in performance between electric nebulizer systems suggests that treatment efficacy may vary greatly in actual patients. The goal of the HPN was to be as close to the ideal as possible, i.e., between 1 and 5 micrometers for particle size as well as having the same flow rate and delivery volume.

To design and build a low-cost nebulizer compressor that provides performance equivalent to the best available devices, but does not require electricity—and would be operated by human effort—levied additional design constraints for performance including operational feedback, user effort, and user perception. Yet performance criteria are not the only considerations relevant to the development and assessment of a device like the HPN; additional design parameters for developing contexts must include considerations of device implementation.

## Conclusions

Respiratory disease remains a leading cause of death and disability in low and middle income countries. Nebulizers are essential tools in the management and diagnosis of respiratory pathology but their use has been limited to areas with access to electricity. The reported data suggest that the HPN’s performance is equivalent to a popular commercially available electric nebulizer compressor. Consequently, the HPN could serve as an important diagnostic and therapeutic tool in the fight against global respiratory health challenges including: chronic obstructive pulmonary disease, asthma, tuberculosis and lower respiratory infections.

## Competing interests

The authors declare that they have no competing interests.

## Authors’ contributions

CJH: conceived of the study, participated in the design of the study, drafted manuscript, performed data collection. MTL: conceived of the study, participated in the design of the study, drafted manuscript, performed data collection. CEZ: participated in the design of the particle sizing studies. WKK: participated in the design of the particle sizing studies. LEO: conceived of the study, participated in the design of the study, drafted manuscript, performed data collection. All authors read and approved the final manuscript.
